# Timing of Initiating Glycopeptide Therapy for Methicillin-Resistant *Staphylococcus aureus* Bacteremia: The Impact on Clinical Outcome

**DOI:** 10.1155/2013/457435

**Published:** 2013-01-20

**Authors:** Chen-Hsiang Lee, Chun-Chih Chien, Jien-Wei Liu

**Affiliations:** ^1^Division of Infectious Diseases, Department of Internal Medicine, Kaohsiung Chang Gung Memorial Hospital, Chang Gung University College of Medicine, Kaohsiung 833, Taiwan; ^2^Department of Clinical Pathology, Kaohsiung Chang Gung Memorial Hospital, Kaohsiung 833, Taiwan

## Abstract

When a *Staphylococcus*-like organism (SLO) is microscopically found in Gram staining of blood culture (BC) specimen, it seems reasonable to administrate a glycopeptide (GP) for empirical therapy. The paper investigates the risk factors for 14-day mortality in patients with methicillin-resistance *Staphylococcus aureus* bacteremia (MRSAB) and clarifies the impact of the timing for initiating GP therapy. A retrospective study identifies patients with MRSAB (endocarditis was excluded) between 2006 and 2009. Patients were categorized as receiving GP at the interval before a preliminary BC report indicating the growth of SLO and the onward 24 hours or receiving GP 24 h after a preliminary BC report indicating the growth of SLO. Total 339 patients were enrolled. There was no difference on the 14-day overall or infection-related mortality rates at the time to administer GP. Multivariate analysis disclosed pneumonia (OR = 4.47; of 95% CI; of 2.09–9.58; *P* < 0.01) and high APACHE II score (OR, 2.81, with 95% CI, 1.19–6.65; *P* = 0.02) were independent risk factors for infection-related mortality. The mortality rate did not decrease following administrating GP immediately after a preliminary BC indicating SLO growth. An additional research for the optimal timing for initiating GP treatment is warranted.

## 1. Introduction

The incidence of methicillin-resistant* Staphylococcus aureus* (MRSA) infections has increased over the past decade [1]. When a *Staphylococcus*-like organism (SLO) is microscopically found in Gram staining of blood culture (BC) specimen, it seems reasonable to administrate a glycopeptide (GP) for empirical therapy. Such practice may lead to antibiotic overuse, while it is uncommon that the SLO eventually turned out to be a contaminant from skin flora [2]. Over-prescribing GPs renders increased adverse events, medical costs, and high GP selective pressure may lead to decreased susceptibility to GP in both methicillin-sensitive* Staphylococcus aureus* (MSSA) and MRSA [3]. However, GP therapy is not always started by some clinicians until they notified the growth of a SLO in a preliminary BC report; as a result, prescription for antibiotic for potential *Staphylococcus aureus* bacteremia may therefore be delayed.

For patients with MRSA bacteremia (MRSAB), how clinical outcomes are affected by differential time of initiating GP therapy remains uncertainty [4–11]. The aims of this study were to elucidate clinical effects for the timing of initiating GP for patients with MRSAB when their preliminary BC report indicated the growth of SLO and to identify risk factors for 14-day overall or infection-related mortality in this patient population.

## 2. Material and Methods

### 2.1. Hospital Setting and How Blood Culture Specimens Were Handled

This is a retrospective study in which the included patients were adults (aged ≥ 18 years) with monomicrobial MRSAB treated with a GP (either vancomycin or teicoplanin) between July 1, 2006 and June 30, 2009 at Kaohsiung Chang Gung Memorial Hospital, a 2500-bed facility serving as a primary care and tertiary referral centre in southern Taiwan. In case an included patient experienced multiple MRSAB, only the first MRSAB episode was counted. We analyzed the participants' demographic and clinical information. The study was conducted with a waiver of patient consent approved by the Institution Review Board of Chang Gung Memorial Hospital, Taiwan (number 95-1249B).

Staff of microbiology laboratory performed Gram staining and subculture of the blood drawn from positive BC bottles alarmed by the incubation machine. As Gram-positive cocci growing in grape-like clusters were found microscopically, a preliminary BC report about SLO was released, and the medical staff would be immediately informed by phone for the result. A formal report was released when the species of the SLO were identified, and antimicrobial susceptibility tests were completed using standard microbiological methods [12, 13]. An MRSA was defined as a tested* S. aureus* against that cefoxitin impregnated in a diffusion disk produced an inhibition zone ≤19 mm [14]. Clinical criteria for true bloodstream infections were as followings: (I) patients with the same species isolated from 2 or more sets of blood cultures. (II) Patients with the same species isolated in 1 of initial 2 sets of blood cultures and additional blood cultures have systemic inflammation reaction syndrome. (III) Patients with a species growing in 1 set of blood cultures, and without an obvious evidence of an infectious source, in the presence of systemic inflammatory response syndrome, had at least one of the following: (1) shock, metabolic acidosis, or disseminated intravascular coagulation; (2) indwelling intravascular devices for more than 48 h, and (3) receipt of hemodialysis or peritoneal dialysis.

### 2.2. Study Design

The severity of the illnesses at the time when sampling the patients' blood for culture was assessed using modified APACHE II score [15] and was stratified based on (i) the acquisition of infection from community or hospital settings [16] and (ii) the need for admission to an intensive care unit (ICU) or not. The APACHE II scoring was modified as follows: zero points were respectively given to the items PaO_2_ and pH if an arterial blood gas analysis was not performed because of the absence of respiratory distress. “Primary bacteremia” defined conditions in which no primary focus could be determined. In the case of secondary bacteremia, a primary focus of infection was determined by the following definitions. The term “pneumonia” was retained in patients with clinical signs of lower respiratory tract infection associated with radiographic evidence of pulmonary infiltrates not attributable to other causes. An intravenous catheter was considered the source of bacteremia if the catheter had been in place for at least 72 h, culture of a quantitative catheter specimen yielded more than 100 colonies of *S. aureus*, or culture of a specimen of purulent drainage from the insertion site grew *S. aureus *[17]. Endovascular source was defined as aneurysms and infection due to vascular grafts or other endovascular devices. Urinary tract infection was considered if the patient had urinary symptoms, and *S. aureus* (>10^5^ colony-forming units per millilitre) was identified as the sole pathogen from urine. Osteomyelitis was defined if *S. aureus* was identified, as the sole pathogen from bone tissue or blood culture yielded *S. aureus*, and the image study (MRI or radionuclide scanning) reveal areas of bone inflammation. Soft tissue infection was considered in the case of patients who had a pure culture of *S. aureus* from a tissue or drainage specimen from the affected site and signs of infection. Endocarditis was considered in patients with *S. aureus* bacteremia and 1 or more of the following characteristics: surgical or autopsy findings consistent with endocarditis, echocardiographic evidence of valvular vegetation, and the presence of septic emboli [18].

As bacteremia due to endocarditis is different from other bacteremia in terms of severity of infection and duration of therapy, MRSA bacteremic patients with endocarditis were excluded in this study. The timing of initiating antibiotic therapy and the dose (vancomycin 15–20 mg/kg every 12 h or teicoplanin 6–12 mg/kg per day) of GP were at the discretion of the patient's physician. For evaluation of the clinical effects of the timing for initiating GP therapy for MRSAB, the included patients were categorized as two groups: received GP at the interval between before a preliminary BC report indicating the growth of SLO and the onward 24 hours or received GP 24 h after a preliminary BC report indicating the growth of SLO. The primary outcome of interest was 14-day overall or infection-related mortality, which was defined as overall or infection-related mortality occurring during the hospital admission in the time period within 14 days since sampling blood for culture.

### 2.3. Statistical Analysis

Categorical variables were compared using the Chi-squared test or Fisher's exact test. Continuous variables were compared using *t*-test or Mann-Whitney *U* test between different groups. Demographic and clinical differences between the deceased and survived patients in comparisons were assessed using univariate analyses. To identify independent risk factors for the 14-day overall or infection-related mortality of MRSA bacteremic patients, variables with a  *P*  value of ≤0.10 from univariate analyses and different time of administering GP therapy (before a preliminary BC report indicating the growth of SLO or for the next 24 h) were separately entered into a multivariate logistic regression model to identify independent risk factors for the 14-day overall or infection-related mortality of MRSAB patients. A 2-tailed *P* value of <0.05 in multivariate logistic regression was considered statistical significance. Statistical analysis was performed with the SPSS software package, version 11.5 (SPSS Inc., Chicago, IL, USA).

## 3. Results

### 3.1. Patient Characteristics

During the study period, 1302 specimens of BC grew SLO, 504 were MSSA, 458 were MRSA, and 340 were coagulase-negative *Staphylococcus*. A total 458 blood MRSA specimens were isolated from 435 patients. After exclusion of patients who died of unrelenting sepsis on the day of admission, those who were discharged from our emergency department on the same visiting day, those who had polymicrobial bacteremia or those who had endocarditis, or those did not received a GP during hospitalization, 339 patients with MRSAB were included for analyses. The mean elapsed time from sampling blood for culture to SLO was 22.8 ± 8.0 h. Prior to starting GP therapies (teicoplanin 83.2% and vancomycin 16.8%) for MRSAB, 245 of the included patients received treatment with antibiotics other than a GP (81.6% received *β*-lactams, 18.3% quinolones, and 10.2% aminoglycosides). A total of 56 (16.5%) patients died within 14 days after SBFC, and 38 (11.2) patients died due to MRSA infection. There was no significant difference in overall or infection-related mortality rates among patients received treatment with teicoplanin or vancomycin (16.3% versus 17.5%; *P* = 0.82 and 10.6% versus 14.0%; *P* = 0.46, resp.).

### 3.2. Factors Associated with Timing of Administration of Glycopeptide Therapy

Univariate analyses of initial GP therapy stratified according to the timing of preliminary BC indicating SLO growth were summarized in Table 1. The significantly different variables between different groups of GP therapy stratified accordingly to the timing of preliminary BC indicating SLO growth included male patients (*P* = 0.03), uremia requiring hemodialysis (*P* = 0.05), admission to ICU (*P* = 0.03), and APACHE II score > 15 (*P* < 0.01). It suggested that clinical severity was the clinicians' main concern and indication for starting GP therapy.

### 3.3. Predicting 14-Day Mortality Associated with MRSAB

Univariate analyses for patients with MRSAB who died within 14 days (*n* = 56) and for survivors (*n* = 283) were summarized in Table 2. Significantly different variables included diabetic mellitus (53.6% versus 37.8%; *P* = 0.04), APACHE II score > 15 (23.2% versus 10.9%; *P* = 0.03), pneumonia (35.7% versus 10.9%; *P* < 0.01), and catheter-related infection (1.8% versus 13.8%; *P* < 0.01). Univariate analyses for patients with MRSAB who died due to infection within 14 days (*n* = 38) and for survivors (*n* = 301) were summarized in Table 3. Significantly different variables included APACHE II score > 15 (26.3% versus 11.3%; *P* = 0.02) and pneumonia (39.5% versus 11.9%; *P* < 0.01). Different interval for initiating GP therapy before the preliminary BC report indicating the growth of SLO and within 24 hours did not significantly affect 14-day overall or infection-related mortality. 

### 3.4. Multivariate Analyses to Identify Risk Factors for 14-Day Mortality Associated with MRSAB

Multivariate analysis disclosed that patients with diabetes mellitus (odds ratio (OR) = 1.97; 95% CI of 1.06–3.68; *P* = 0.03), infection site of catheter (OR = 0.13; 95% CI of 0.02–0.99; *P* = 0.05), infection site of lung (OR = 3.95; 95% CI of 1.98–7.91; *P* < 0.01), and APACHE II score > 15 (OR = 2.24; 95% CI of 1.02–4.89; *P* = 0.04) were independent risk factors for 14-day overall mortality in patients with MRSAB (Table 4). It also disclosed that infection site of lung (OR = 4.47; 95% CI of 2.09–9.58; *P* = 0.02), and APACHE II score > 15 (OR = 2.81; 95% CI of 1.19–6.65; *P* = 0.02) were independent risk factors for 14-day infection-related mortality in patients with MRSAB (Table 4).

## 4. Discussion

Early administration of GP therapy should concern emerging resistance due to widespread use of GPs [8], possible suboptimal therapies for MSSA infections [19, 20], the rising incidence of MRSA infection [21], and potential additional morbidity associated with delaying appropriate treatment [3]. The relationship between timing of effective antibiotics administration and outcomes has resulted in conflicting conclusions between numerous studies exploring mortality predictors for MRSAB [6–11, 22]. These discrepancies may be due to different definitions of the timing for appropriate antibiotic therapy, dosing of GP administration, analysis methods, adjustment difficulties, and diverse patient populations. Among which, some of patients started GP treatment, and others switched to GPs after receiving *β*-lactams, while many received other antibiotic therapy. This study indicated that discordant therapy (initial no GP therapy for MRSA infection) is not the only factor determining mortality. Consistent with previous reports [9–11], our results also illustrated that initiating GP therapy earlier, after a positive preliminary BC, did not reduce the 14-day mortality of patients with MRSAB.

The guidelines for the prophylaxis and treatment of MRSA infections in the UK suggested that when the strain is oxacillin susceptible, step-down therapy, shifting from an agent encompassing MRSA to oxacillin, is safer than its alternative, the escalation therapy [23]. However, this suggestion was not supported by definitive clinical studies, epidemiological studies, or a theoretical rationale. GPs, rather than *β*-lactam therapy, might have resulted in higher rates of relapse and mortality in patients with MSSA bacteremia [19, 20, 22]. Emerging vancomycin resistance among Gram-positive organisms is a major threat to patient's safety in hospitals, and overusing GPs could promote the selection and spread of these resistant organisms [24]. Clinicians should prudently prescribe GPs, and the decision for empirical GP therapy requires additional data, such as a rapid polymerase-chain-reaction BC test for MRSA [25].

Except severity of MRSAB represented by high APACHE II score, concurrent pneumonia was an independent risk factor for our MRSA bacteremic patients with regard to 14-day all-cause and attributed mortalities. As is known, GPs have poor penetration into lung tissues [26], and standard vancomycin doses may be subtherapeutic in critically ill patients [27]. Linezolid, which has a greater lung penetration rate and better pharmacokinetic properties [28], might be the therapeutic choice for these particular patients.

This study was performed at a single center, and the results may not be generalized to the outside of this population, especially to areas with a high prevalence of MRSAB. The nature of this observational study was a limitation; a randomized controlled trial assessing the effects of appropriate therapy is neither ethical nor feasible. The result lacks of dosing regimens of GP administration and lacks of GP target attainment. While the efficiency of GP therapy on the isolates was not assessed with minimum inhibitory concentration (MIC) or serum concentration data for vancomycin or teicoplanin. Patients with MRSAB who were treated with vancomycin had a higher risk of treatment failure and mortality when the isolate MIC was >1 mg/L [29]. No data existed regarding to the serum concentration of teicoplanin, and general infections may have required >10 mg/L and endovascular infections >20 mg/L [30]. We did not take into account susceptibility/resistance to non-GP drugs (particularly fluoroquinolones and aminoglycosides). Nevertheless, the fluoroquinolones and aminoglycosides are suboptimal therapies for MRSA infection [31]. Finally, we excluded MRSA bacteremic patients with endocarditis due to endocarditis that is different from other MRSAB in terms of severity of infection and needed for aggressive surgical intervention. However, measuring 14-day mortality may be well-represented therapeutic effect for nonendocarditis bloodstream infection.

## 5. Conclusions

We found that deciding when to initiate GP therapy must take into account of the expected clinical benefits to the individual patient against the public health implications of overusing GPs. Early and precise methods are needed to predict *S. aureus* infection and methicillin susceptibility for appropriate empirical therapy for MRSA. We here recommended additional research to establish the optimal timing for initiating GP treatment in patients with MRSAB.

## Figures and Tables

**Table 1:** Factors affecting when GP therapy was initiated in patients with MRSA bacteremia.

Variable	GP therapy was initiated and stratified according to the timing of preliminary BC indicating SLO growth, No. (%)		*P*-value
	Before and within 24 h	After 24 h	
No. of patients	191 (56.2)	148 (43.8)	
Age in years, median (range)	62 (28–98)	63 (25–88)	0.89
Male gender	111 (58.1)	103 (69.6)	0.03
Underlying disease or condition			
Cardiovascular disease	26 (13.6)	23 (15.5)	0.64
Diabetes mellitus	76 (39.8)	61 (41.2)	0.82
Uremia requiring dialysis	30 (15.7)	36 (24.3)	0.05
Decompensated liver cirrhosis	32 (16.8)	15 (10.1)	0.08
Malignancies	32 (16.8)	32 (21.6)	0.26
Prosthetic device implantation	21 (10.9)	20 (13.5)	0.51
Severity-of-illness marker^a^			
Nosocomial acquisition	141 (73.8)	104 (70.3)	0.54
Intensive care unit admission	51 (26.7)	24 (16.2)	0.03
APACHE II score, median (range)	20 (7–30)	17 (2–24)	0.07
APACHE II score > 15	34 (17.8)	10 (6.8)	<0.01
Source of infection^b^			
Catheter-related infection	18 (9.4)	22 (14.9)	0.13
Endovascular infection	3 (1.6)	6 (4.1)	0.19
Soft-tissue infection	55 (28.8)	43 (29.1)	1.00
Osteomyelitis	44 (23.6)	23 (15.5)	0.10
Urinary tract infection	1 (0.5)	3 (2.0)	0.32
Pneumonia	35 (18.3)	16 (10.8)	0.07
Primary bacteremia	46 (24.1)	28 (18.9)	0.29

BC: blood culture; GP: glycopeptide; MRSA: methicillin-resistant *S. aureus*; No.: number.

^a^At time of blood culture sampling.

^b^Some patients had more than one infected site.

**Table 2:** Variables associated with 14-day overall mortality.

Variable	Yes, No. (%)	No, No. (%)	*P-*value
No. of patients	56 (16.5)	283 (83.5)	
Age in years, median (range)	68 (38–98)	65 (22–93)	0.45
Gender, male	33 (58.9)	181 (63.9)	0.55
Underlying disease/condition			
Cardiovascular disease	10 (17.9)	39 (13.8)	0.41
Diabetes mellitus	30 (53.6)	107 (37.8)	0.04
Uremia requiring dialysis	10 (17.9)	56 (19.8)	0.85
Decompensated liver cirrhosis	10 (17.9)	37 (13.1)	0.40
Malignancies	9 (16.1)	55 (19.4)	0.71
Prosthetic device implantation	6 (10.7)	35 (12.4)	0.83
Severity-of-illness marker^a^			
Nosocomial acquisition	46 (82.1)	199 (70.3)	0.07
Intensive care unit admission	16 (28.6)	59 (20.8)	0.22
APACHE II score, median (range)	18 (1–31)	17 (2–38)	0.19
APACHE II score > 15	13 (23.2)	31 (10.9)	0.03
Source of infection^b^			
Catheter-related infection	1 (1.8)	39 (13.8)	<0.01
Endovascular infection	0	9 (3.2)	0.37
Soft-tissue infection	10 (17.9)	79 (27.9)	0.42
Osteomyelitis	12 (21.4)	55 (19.4)	0.72
Urinary tract infection	0	4 (1.4)	1.00
Pneumonia	20 (35.7)	31 (10.9)	<0.01
Primary bacteremia	10 (17.9)	64 (21.8)	0.48
Timing of initiating glycopeptide therapy			
Before preliminary BC report indicated SLO growth and within 24 h after BC indicated SLO growth	36 (64.3)	155 (54.8)	0.24

BC: blood culture; MRSA: methicillin-resistant *S. aureus;* No.: number; SLO: *Staphylococcus*-like organism.

^a^At time of blood culture sampling.

^b^Some patients had more than one infected site.

**Table 3:** Variables associated with 14-day infection-related mortality.

Variable	Yes, No. (%)	No, No. (%)	*P-*value
No. of patients	38 (11.2)	301 (88.8)	
Age in years, median (range)	68 (38–90)	64 (23–88)	0.54
Gender, male	24 (63.2)	190 (63.1)	1.00
Underlying disease/condition			
Cardiovascular disease	7 (18.4)	42 (13.9)	0.46
Diabetes mellitus	20 (52.6)	117 (38.9)	0.12
Uremia requiring dialysis	7 (18.4)	59 (19.6)	1.00
Decompensated liver cirrhosis	6 (15.8)	41 (14.6)	0.80
Malignancies	7 (18.4)	57 (18.9)	1.00
Prosthetic device implantation	5 (13.2)	36 (11.9)	0.79
Severity-of-illness marker^a^			
Nosocomial acquisition	31 (81.6)	214 (71.1)	0.25
Intensive care unit admission	12 (31.6)	63 (20.9)	0.15
APACHE II score, median (range)	15 (1–38)	11 (2–29)	0.15
APACHE II score >15	10 (26.3)	34 (11.3)	0.02
Source of infection^b^			
Catheter-related infection	1 (2.6)	39 (12.9)	0.07
Endovascular infection	0	9 (2.9)	0.61
Soft-tissue infection	13 (34.2)	85 (28.2)	0.45
Osteomyelitis	8 (21.1)	59 (19.6)	0.83
Urinary tract infection	0	4 (1.3)	1.00
Pneumonia	15 (39.5)	36 (11.9)	<0.01
Primary bacteremia	6 (15.8)	68 (22.6)	0.41
Timing of initiating glycopeptide therapy			
Before preliminary BC report indicated SLO growth and within 24 h after BC indicated SLO growth	23 (60.5)	168 (55.8)	0.61

BC: blood culture; MRSA: methicillin-resistant *S. aureus;* No.: number; SLO: *Staphylococcus*-like organism.

^a^At time of blood culture sampling.

^b^Some patients had more than one infected site.

**Table 4:** Multivariate analysis of risk factors for 14-day mortality.

Variable	*P-*value	OR (95% CI)
Overall mortality		
Diabetes mellitus	0.03	1.97 (1.06–3.68)
Nosocomial acquisition	0.17	1.71 (0.80–3.69)
Catheter-related infection	0.05	0.13 (0.02–0.99)
Pneumonia	<0.01	3.95 (1.98–7.91)
APACHE II score > 15	0.04	2.24 (1.02–4.89)
Timing of initiating glycopeptide therapy		
Before preliminary BC report indicated SLO growth and within 24 h after BC indicated SLO Growth	0.40	1.37 (0.66–2.84)
Infection-related mortality		
Catheter-related infection	0.16	0.23 (0.03–1.78)
Pneumonia	<0.01	4.47 (2.09–9.58)
APACHE II score > 15	0.02	2.81 (1.19–6.65)
Timing of initiating glycopeptide therapy		
Before preliminary BC report indicated SLO growth and within 24 h after BC indicated SLO Growth	0.90	0.95 (0.41–2.21)

BC: blood culture; MRSA: methicillin-resistant *S. aureus;* SLO: *Staphylococcus*-like organism.
